# Book Review

**Published:** 1899-06

**Authors:** 


**Elements of Histology.** By E. KLEIN, M.D., F.R.S., and J. S. EDKINS, M.B. Revised Edition. Pp. xii., 500. London: Cassell and Company, Limited. 1898.—Although it is not so stated on the title-page, this is the third edition of this useful manual, and between its first appearance in 1883 and the present issue there have been ten reprints. It is quite modern; for many additions have been made, especially in the sections treating of the cell and the nervous system. The improved methods of staining introduced by Golgi and others have made it necessary to introduce numerous fresh illustrations and diagrams. Only eighteen pages are given to the cell, but a great deal of new matter has been introduced, and the chapter may be taken as a fair *résumé* of the present knowledge of the subject. The somewhat puzzling terms that have been manufactured of late to express the different parts of the cell-structure and the changes in the nucleus, etc., are clearly used; but there is some redundancy—*e.g.* mitoma, intra-nuclear network, and chromosomes, all meaning the same. A noticeable feature is the photo-micrographs which appear in this edition. Some are useful; but many are very poor, especially those on pages 57, 205, 301, and 332. These are so indistinct as to be almost worthless. On the other hand, most of the illustrations and diagrams are decidedly good, and the account of the histology of the various organs is concise and clear; so that an average student can gain an excellent knowledge of minute structure from this work, which is sure to be popular.

**Practical Organic Chemistry.** By S. RIDEAL, D.Sc. Second Edition. Pp. x., 172. London: H. K. Lewis. 1898.—The second edition of this text-book has afforded the author an opportunity of including some additions to the lists of substances, a practical acquaintance with which is required of candidates for certain of the higher examinations. The properties and reactions of the more common organic bodies are concisely and accurately described, and the information is conveyed in a manner which will render it easily assimilable by those to whom it is especially addressed.

**Notes on Malaria in connection with Meteorological Conditions at Sierra Leone.** By Surgeon-Major E. M. WILSON. Second Edition. Pp. 16. London: H. K. Lewis. 1898.—We reviewed the first edition of this booklet some time ago, and pointed out the salient features of the opinions of the author. We have not yet heard that the Government have made the alteration in diet Major Wilson recommends. But then permanent officials move slowly.

**Materia Medica, Pharmacy, Pharmacology, and Therapeutics.** By W. Hale White, M.D. Third Edition. Pp. xvi., 621. London: J. & A. Churchill. 1898.—Except to record that this third edition of Dr. Hale White's work is fully brought up to date and includes all the changes in the recent edition of the *British Pharmacopœia*, it is not necessary to enter into any detailed criticism upon it. It remains, as before, admirably concise, containing all that it is absolutely essential for a student to know about pharmacology and the therapeutical uses of drugs. Although treating mainly of substances included in the *British Pharmacopœia*, the book is not exclusively restricted thereto, for many useful but non-official remedies, such as exalgine, are noticed in their proper places. We heartily commend the work.

**Notes on Pharmacy and Dispensing for Nurses.** By C. J. S. Thompson. Pp. 101. London: The Scientific Press, Limited. 1898.—Nurses will find this small work of use in learning how to dispense medicines. The author has put what he wants them to learn clearly and as briefly as possible, but he has sometimes rather outstepped the range of what a dispenser has to do.

**Baby Feeding.** By A Doctor. Pp. 62. Bristol: John Wright & Co. 1898.—The information given by the author, who modestly appears as M.B., B.Ch. (Univ. Dub.), is of a thoroughly sound nature, clearly and forcibly expressed. We are afraid, however, there are a few "nots" in Chapter VI. that are seldom attended to, and, moreover, never will be as long as the world goes round. For all that, the advice is good and cannot be too often repeated. Drops of water wear the rock, and we wish every success to the author's drops on the rocks of superstition and ignorance.

**Health Loss and Gain.** No. 1. By M. A. Chreiman. Pp. 226. London: The Rebman Publishing Company, Limited. 1898.—The author proposes "that there should be instituted, as a custom, a system of periodical examination, to which all persons should submit themselves, and to which they should submit their children." We fear that his method would be an excellent means for the multiplication of the nervous hypochondriac, and we hold that if apparent health is working duly without friction it is best to leave it so. The writer has views on many things, and the volume is well worthy of perusal; but one cannot fail to observe that there is an absence of the medical instinct usually associated with the medical mind.

**Dwelling Houses: their Sanitary Construction and Arrangements.** By W. H. Corfield, M.D. Fourth Edition. Pp. xiv., 125. London: H. K. Lewis. 1898.—This edition has been carefully revised throughout by the author, and within its scope forms a good guide for the lay reader.

**Association Française de Chirurgie. Onzième Congrès. Procès-verbaux, Mémoires et Discussions.** Paris: Félix Alcan. 1897.—A large field is covered in these transactions; but especial attention should be directed to the papers and discussions on contusions of the abdomen, and the operative treatment of cancer of the rectum. The book is suitably illustrated in various parts, and reflects the highest credit on the work of French surgeons.

**Recherches cliniques et thérapeutiques sur l'Épilepsie, l'Hystérie, et l'Idiotie.** Vol. XVIII. Paris: Félix Alcan. 1898.—In the Bicêtre Asylum, of which this is the annual report there is excellent work being done. Records of many interesting cases of idiocy are given, amongst which are several of importance from a pathological point of view, and of great interest as regards the lesions of the brain. Dr. Bourneville finds in one case a further confirmation of his views as to false and true porencephaly, and it would certainly be well if the present vague use of this term was given up for a more restricted signification. A case in which there were lesions of both frontal lobes is also worthy of study. We must call especial attention to the beautiful reproductions of photographs of the brains of the most important cases, which are given in a series of fine plates, eighteen in number. We regard this volume as an important contribution to the pathology of the brain conditions underlying some forms of idiocy, and the cases recorded in it are worthy of careful perusal.

**King's College Hospital Reports.** Vol. IV. London: Adlard and Son. 1898.—This volume contains a continuation of John Curnow's historical sketch, and has an interesting account of the enthusiasm and the work of Robert Bentley Todd. The original papers are not numerous: Dr. Nestor Tirard gives an interesting account of albuminuria in children; Mr. Albert Carless advocates early operation in cases of appendicitis; Dr. Arthur Whitfield, writing on the diagnosis and management of syphilis, advocates the view "that iodide may be used to help in the improvement of some symptoms, but on mercury must we rely wherewith to combat the disease"; Dr. Raymond Crawfurd prefers a combination of belladonna and nux vomica to various barbarities which have been reputed to be of use for the enuresis of childhood; Dr. Hugh Playfair considers it to be absolutely essential to combine local application with constitutional treatment for most cases of leucorrhœa; and Dr. J. Curtis Webb gives some interesting reminiscences of gynæcology in Berlin. The statistical reports and records of cases of interest follow, and the account of what old King's men are doing is of especial interest to those whose names are found there.

**Saint Bartholomew's Hospital Reports.** Vol. XXXIII. London: Smith, Elder, & Co. 1898.—The volume of reports begins this year, as it has unfortunately done rather often of late, with an obituary notice of a member of the hospital staff. In the present volume Dr. Church gives a very interesting account of the life of Dr. James Andrew, who retired from work at the hospital some few years ago, and died in April, 1898. Such accounts are of sad but special interest to all old Bartholomew's students, and the graphic description of Dr. Andrew in his ward work is one which will recall many scenes connected with their study of clinical medicine. There are many papers of great value in the reports this year. As we might expect, many record interesting cases which have been in the hospital, and both the clinical and pathological sides of the work have yielded important results. There is perhaps no record of clinical work which gives so accurate an account of the difficulties and mistakes in diagnosis, and the failures in treatment, as the report of all cases of interest occurring in a large hospital during the year, and these we find admirably given in this volume. Instead of the publication of the successful cases only, to which we are all tempted, we have here many examples of failure both in diagnosis and treatment, which are really often more instructive than the successful ones.

**The American Year-Book of Medicine and Surgery.** London: The Rebman Publishing Co. (Ltd.). Philadelphia: W. B. Saunders. 1899.—*The American Year-Book*, a digest collected and arranged by a large staff under the general editorial charge of Dr. George M. Gould, has now become an established perennial in its fourth year. The volume covers a large scope, including all the special branches of medicine and surgery, pharmacology, anatomy, physiology, legal medicine, public hygiene and preventive medicine. Sixteen of the twenty-eight contributors are well-known specialists and teachers of Philadelphia; New York city claims four, Chicago three, Cleveland two; and Baltimore, Boston, and Montreal can each claim one. The staff may well be complimented on "that expertness of intelligence in gleaning and ripeness of judgment in deciding as to values which can only be gained by experience and knowledge." The book may be opened at any page, and the reader will find something interesting, novel, and well presented in excellent type. The omission of the name of Dr. William Pepper from the list of contributors is mentioned by the editor, and the whole world of medicine is the poorer by the death of that distinguished man.

**Annual and Analytical Cyclopædia of Practical Medicine.** Vol. II. The F. A. Davis Company. 1899.—The second volume of this great work has now arrived. It commences with bromide of ethyl and concludes with diphtheria. The volume contains exceptionally valuable articles on a number of exacting subjects, viz.: "Cerebral Hæmorrhage," by Dr. William Browning, of Brooklyn; "Cirrhosis of the Liver," by Professor Adami, of Montreal; "Cholera," by Professor Rubino, of Naples; "Cholelithiasis," by Professor Graham, of Toronto; "Diabetes," by Professor Lépine, of Lyons; "Diphtheria," by Drs. Northrup and Bovaird, of New York; "Constipation," by Professor Nathan S. Davis, of Chicago; "Dilatation of the Heart," by Dr. Vickery, of Boston. These and many other articles deserve the reader's special attention and study. The volume is beyond all praise or criticism, and Dr. Sajous deserves the hearty thanks of all engaged in the practice of medicine.

**The Medical Annual.** Bristol: John Wright & Co. 1899.—The amount of useful information given to the busy man in this volume is remarkable. Most of the contributors to it are not mere abstractors, but are experienced in various branches of medicine and surgery, and so are able to select from and to criticise the great mass of newly-published work which they lay under contribution. The book is, as has hitherto been the case, well printed and illustrated, and we cordially recommend it to our readers. We think that many provincial societies deserve a place in the list of "Medical and Scientific Societies."

**The Edinburgh Medical Journal.** New Series, Vol. IV. Edinburgh: Young J. Pentland. 1898.—The half-yearly volume contains a collection of excellent papers worthy of careful study by those who have not received the monthly parts. Many of the papers read at, and a general summary of the proceedings of, the British Medical Association at Edinburgh are included. Dr. Finlayson has an interesting paper on "Medical Bibliography and Medical Education," in which he advocates the more general teaching of medicine by clinical work, and cites the instance of Johns Hopkins University, which has abolished systematic lectures on medicine. The reviews of British and foreign literature, and the reports on recent advances in medical science, are done with much care and by authorities in the various departments. We congratulate the editor on the result of his efforts.

Ἰατρικὴ Πρόοδος. *La Grèce Médicale.* Syra: Impr. Renieri Brindesi.—Medical journalism in Greece has had apparently rather a chequered career, one or two journals which we have seen in past years being now extinct. Ἰατρικὴ Πρόοδος is, however, in the fourth year of its existence, and has hitherto appeared in the Greek language only. In order, however, that the active advance of medicine in Greece may become more generally known among other nations, with the monthly issue of the Greek journal there is now published an appendix in French, *La Grèce Médicale.* Of both sections we desire to speak with much commendation; the subject-matter and occasional illustrations are thoroughly praiseworthy, and we wish this addition to our exchange-list long life, and its editor, Dr. Foustanos, a full measure of success.

**Archives provinciales de Médecine.** Paris: Institut international de Bibliographie scientifique.—We are glad to welcome on our exchange-list this new monthly journal, which, following somewhat on the lines of the *Archives provinciales de Chirurgie*, has started a career in the direction of decentralisation in medicine. With a large staff of supporters scattered throughout the principal cities of France, it may be expected to have a very successful and useful course, and we wish Dr. Marcel Baudouin, the editor, all possible recognition of this new effort which he is making, in addition to those many others which have so considerably benefited the profession.

**The British Food Journal.** Vol. I., No. 1. January, 1899. London: Baillière, Tindall & Cox.—This is one of the most remarkable—we had almost said impudent—pseudo-scientific publications that has ever appeared in print. The responsibility for its existence rests upon the shoulders of an "editorial staff" including some names which it is to be hoped decorate the cover without the knowledge of their owners. After drawing attention to certain excellent recommendations by the Censors of the Institute of Chemistry on the question of unprofessional conduct, with special reference to advertising and the issue of trade puffs, the "editorial staff" proceed to traverse the more important ones by offering themselves to the manufacturing world as testimonial-mongers on the co-operative system, on the principle, it is to be supposed, of the unkickability of the corporate body. The fact that this proposal is enshrined in a quantity of doubtful information collected apparently from the columns of the daily papers does not alter its character; and it is much to be desired that the Institute may use its influence to check a movement that can only tend to discredit one of the youngest, but not least useful, of the professions.

**The Philadelphia Monthly Medical Journal.**—Dr. Gould's excellent weekly journal is now to have a monthly supplement, which is to consist of original contributions only. For very good reasons no doubt, No. 3 is the first to be issued. It contains the prize essays in connection with the weekly journal. The first prize in the department of medicine was awarded to a paper by Dr. Henry Wald Bettman, of Cincinnati. It describes the shape, position and displacements of the stomach, and gives an excellent account of the diagnosis, prophylaxis, and treatment of gastroptosis and gastro-enteroptosis. A very complete bibliography is appended, and an analysis of the most important recent papers on the subject: on the authority of Meinert[1] we are informed that "every dress fastened about the waist worn by a girl before her fifteenth year leads inevitably to gastroptosis"; there is, therefore, no need to wonder why it is that 90 per cent. of adult women have gastroptosis, whereas it is present in only about 5 per cent. of men. The other papers

[1] *Centralbl. f. innere Med.*, 1896, xvii. 297, 321.

are worthy of careful consideration, and this new departure of Dr. Gould's deserves to be heartily supported.

**The Polyclinic.** Vol. I. No. 1. May, 1899. London: H. K. Lewis.—Our Exchange-list has received the addition of this Journal of the new Medical Graduates' College. Sir W. H. Broadbent writes on "The Necessity for a Medical Graduates' College;" Mr. Jonathan Hutchinson gives "Some Account of the Formation and Aims of the College;" and Dr. Wm. Miller Ord, in considering "The Medical Practitioner as a Student," says: "The scheme of Post-Graduation Teaching now established will be found to offer large opportunities of self-improvement along many lines of work. The whole plan is framed to insure practical instruction in all departments. Those who will occupy the position of teachers, and those who come for instruction, will work side by side assuredly to the benefit of both. . . . The proverb 'Docendo discimus' is as true as ever." The list of officers and Council is one which will command success.

